# Advancing Intervertebral Disc Biology via Omics: Implications for Nucleus Pulposus Progenitor Cell‐Based Regeneration

**DOI:** 10.1002/jsp2.70130

**Published:** 2025-10-16

**Authors:** Anja Stirnimann, Leon Schlagenhof, Benjamin Gantenbein, Fabian Ille

**Affiliations:** ^1^ Center of Competence in Aerospace Biomedical Science and Technology, Lucerne University of Applied Sciences and Arts Hergiswil Switzerland; ^2^ Tissue Engineering for Orthopaedics & Mechanobiology, Bone and Joint Program, Department for BioMedical Research (DBMR), Faculty of Medicine University of Bern Bern Switzerland; ^3^ Department of Orthopaedic Surgery and Traumatology, Inselspital Bern University Hospital, Faculty of Medicine, University of Bern Bern Switzerland; ^4^ Graduate School for Cellular and Biomedical Sciences (GCB), University of Bern Bern Switzerland

**Keywords:** intervertebral disc regeneration, multi‐omics, nucleus pulposus progenitor cells, RNA sequencing, TIE2

## Abstract

**Background:**

Intervertebral disc (IVD) degeneration (IDD) contributes to global disability and involves incompletely understood molecular processes. Recent advances in omics technologies help to unravel the complex biology of IDD and develop novel therapies.

**Methods:**

This narrative review explores how omics approaches—particularly RNA sequencing—have advanced our understanding of IVD biology and how these findings contribute to leveraging the intrinsic regenerative potential of the IVD, with a specific focus on nucleus pulposus progenitor cells (NPPCs). Relevant literature addressing transcriptomic, genomic, proteomic, metabolomic, and epigenetic data, as well as emerging mult‐iomics approaches, was summarized.

**Results:**

Single‐omics studies have provided insight into cellular heterogeneity, gene expression changes, genetic susceptibility loci, proteomic changes, metabolic alterations, and epigenetic regulation. Further, they have highlighted key pathways associated with extracellular matrix remodeling, inflammation, and progenitor cell depletion in IDD. Inconsistencies between *TEK* (the gene encoding TIE2) mRNA levels and TIE2 (Angiopoietin‐1 receptor) protein expression, discussed in this review, emphasize the limitations of single‐omics analyses and underscore the need for multi‐omics studies, which are currently underrepresented in the field. By enabling a system‐level understanding, multi‐omics offers a comprehensive framework to decode the networks driving IDD and identify biomarkers and therapeutic targets to restore disc function and reduce pain.

**Conclusions:**

Although NPPCs hold promise for IVD regeneration, translational challenges such as cell survival and efficacy persist. Omics‐informed insights into the IVD microenvironment may support the development of combinatorial strategies, including co‐delivery of modulators to enhance NPPC survival and the effectiveness of therapies.

## Graphical abstract

Intervertebral disc degeneration (IDD) is a multifactorial condition underpinned by complex molecular mechanisms. This review highlights advances in RNA sequencing and multi‐omics approaches that have elucidated gene regulatory networks, cellular heterogeneity, and progenitor cell dynamics in the nucleus pulposus. Integrated analyses reveal key pathways in matrix remodeling, inflammation, and TIE2‐associated signaling, offering mechanistic insights into regenerative strategies and therapeutic target discovery.

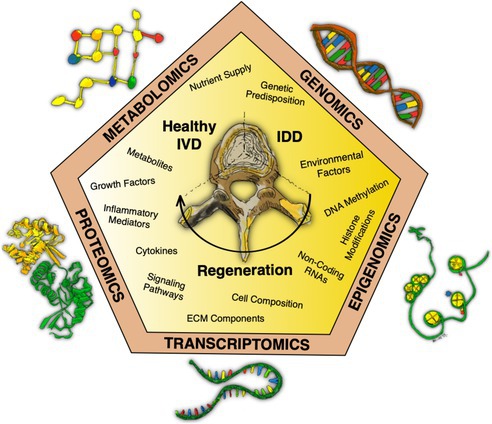

## 1 Introduction

Intervertebral disc (IVD) degeneration (IDD) affects millions of people globally, contributing to low back pain (LBP) and disability. The precise mechanisms underlying IDD remain poorly understood [1]. Advances in omics technologies are beginning to elucidate these complex processes [2, 3]. This review explores how omics technologies have advanced the understanding of the intrinsic regenerative potential of the IVD, with a particular focus on nucleus pulposus progenitor cells (NPPCs). While “intrinsic” traditionally refers to endogenous repair without external cell delivery, our use of the term emphasizes leveraging the disc's native regenerative capacity by isolating and utilizing IVD‐resident cells. Although applied in an extrinsic manner through cell therapy, this approach aims to harness the inherent regenerative properties of endogenous cell populations to restore IVD structure and function. Thus, “regeneration” refers to strategies to restore IVD structure and function—primarily through leveraging the intrinsic repair capacity of resident cells as cell therapy and through endogenous repair mechanisms. In addition, the broader cellular landscape and molecular pathways uncovered through omics studies are discussed, as these factors directly impact NPPC viability, function, and therapeutic efficacy, and co‐modulation might be necessary for effective cell therapy. While progress has been made, significant gaps remain in our knowledge of the molecular pathways involved in IVD degeneration and regeneration, underscoring the need for integrative multi‐omics strategies to inform future therapeutic developments.

### 1.1 The IVD and the Pathophysiology of IVD Degeneration

The IVD is a mostly avascular tissue between adjacent vertebrae in the spinal column, providing mechanical support, shock absorption, and flexibility. It consists of the gelatinous nucleus pulposus (NP) in the center, the fibrous annulus fibrosus (AF) surrounding the NP, and the semi‐permeable cartilage endplates (CEPs) [4]. Together, these structures maintain the function of the spinal column by absorbing load, preventing the vertebrae from grinding against each other, and providing flexibility. Each structure on its own is crucial for IVD function and spinal health. Dysfunction in any component often leads to the onset and progression of IDD.

IDD is a prevalent musculoskeletal disorder involving biological and structural changes in the IVD [5]. Characteristics include NP dehydration, AF disruption, and reduction of proteoglycan content, cellularity, and CEP cell density [6], impairing the IVD function. While asymptomatic degenerative changes are common [7], IDD is recognized as a major contributor to chronic LBP, which imposes a substantial socioeconomic burden [8].

Genetic and environmental factors like age, nutrition, inflammation, mechanical stress, and structural damage [9, 10] contribute to the degenerative process. Chronic LBP associated with IDD leads to reduced productivity, increased healthcare costs, and diminished quality of life for affected individuals [1].

Despite this impact, effective treatments for IDD and associated LBP remain elusive. Current treatment strategies focus on symptomatic relief through physical therapy and pharmacotherapy or surgical interventions, such as spinal fusion or disc replacement [11]. However, these approaches often provide only temporary relief [12, 13] and address the symptoms without targeting the underlying pathological cause of IDD [14]. The multi‐factorial nature of IDD and the limitations of current treatment strategies require more targeted therapies that address the molecular causes of the pathophysiology in IDD, not only pain management. To develop and evaluate such interventions, animal models play an important role.

### 1.2 Animal Models

Animal models are essential in IVD research as they offer physiologically relevant systems to study disease mechanisms and therapeutic interventions with greater experimental control and accessibility than human tissues. Each animal model has distinct advantages and limitations. Rodents offer genetic manipulability and cost‐efficiency but are limited by persistent notochordal cells (NCs), small disc size, and differing mechanical loading. Rabbits are more anatomically relevant with a larger disc size but similarly retain NCs. Chondrodystrophic dogs develop spontaneous disc degeneration with early NC loss, closely mimicking human pathology. Large animal models like goats, sheep, and bovines exhibit similar dimensions, biomechanics, and postnatal NC loss, making them suitable for preclinical testing. Pigs resemble humans in disc size but retain NCs, reducing translational relevance. Nonhuman primates most closely replicate human IVD pathology, including spontaneous degeneration and upright posture, but are constrained by ethical concerns. Model selection, therefore, depends on the specific research objective, balancing anatomical and biological relevance with ethical feasibility [15].

Among large animal models, the bovine coccygeal IVD is frequently used due to its close histological, anatomical, and biochemical resemblance to the human IVD [16, 17]. These similarities include disc size, biomechanics, cell composition, postnatal loss of NCs, and expression of tissue‐specific markers [11, 17, 18, 19]. As a result, it is a widely accepted model system in IVD research [16, 20, 21].

In contrast, rodent models are often used to investigate the genetic and molecular characteristics of IVD development [3]. Their compatibility with high‐throughput molecular tools and genomic resources makes them suited for omics‐based studies. Despite their anatomical limitations, rodent models contribute significantly to elucidating pathways involved in IVD homeostasis and degeneration.

This review focuses on human, bovine, and rodent models, which collectively provide the most comprehensive and accessible datasets for omics analyses. Bovine and rodent models offer complementary advantages for molecular studies, while human data are essential for clinical translation. Leveraging these models enables investigation of the intrinsic regenerative potential of the IVD.

### 1.3 Concept of an Intrinsic Regenerative Potential

The IVD is thought to possess an intrinsic regenerative potential. This term refers to the tissue's ability to maintain and restore its structure and function through resident cells and endogenous repair mechanisms.

Among these cells, NPPCs—a subpopulation of progenitor cells—present in the NP, are thought to play a key role in IVD development, growth, and homeostasis [19, 22]. With progressive age, the cellular composition of the NP changes. NPPCs and NCs reduce with aging and degeneration, coinciding with a diminished regenerative potential [3, 23, 24]. NPPCs display stem cell‐like properties, including self‐renewal, tri‐lineage differentiation potential, and stem cell marker gene expression [25, 26, 27]. Additionally, they influence ECM production and regulate signaling pathways [28, 29], making them key candidates for regenerative strategies.

Since the discovery of TIE2+ NPPCs (NPPCs^TIE2+^) [29], TIE2 (Angiopoietin‐1 receptor protein) has become a key marker for the identification and isolation of NPPCs, and research on NPPCs^TIE2+^ has progressed, recently focusing primarily on their clinical relevance and application [30]. However, discrepancies between *TEK* (the gene encoding TIE2) mRNA levels and TIE2 protein expression levels, as well as inconsistent nomenclature, remain unresolved. Various markers have been suggested for identifying NPPCs, and emerging evidence indicates the existence of different NPPC subsets within the NP. Additionally, NPPC isolation and expansion methods risk altering cell phenotype and differentiation status [25, 29, 31, 32, 33, 34], and sorting technologies, such as fluorescence‐activated cell sorting (FACS), may affect cell integrity [35], necessitating improved techniques for clinical applications. A systematic perspective addressing their microenvironment and regulatory network is needed to overcome these challenges and fully realize the therapeutic potential of NPPCs for IDD treatment.

### 1.4 Necessity of a Systematic Perspective

To fully harness the regenerative potential of NPPCs, it is essential to adopt a systematic perspective encompassing the broader cellular landscape and molecular pathways driving disc degeneration, as these factors directly impact NPPC viability, function, and therapeutic success. NPPCs do not function in isolation but are influenced by interaction with neighboring cells and their local microenvironment. Tissue‐wide changes such as ECM degradation, hypoxia, inflammation, and osmolarity shifts impact the NPPC niche [36]. Signaling pathways such as wingless‐type MMTV integration site family (Wnt), Transforming Growth Factor Beta (TGF‐*β*), and nuclear factor kappa B (NF‐*κ*B) affect the broader environment and thus also NPPCs [37, 38, 39].

With current omics studies analyzing whole IVD or NP tissue, rather than isolated NPPCs, understanding community effects and the NPPC cell niche is challenging, if not impossible. Therefore, interpreting NPPC biology necessitates a comprehensive understanding of the cell niche on top of the global molecular changes.

Finally, successful clinical translation of NPPC‐based therapies will require targeting NPPCs, conditioning the surrounding niche, and modulating the pathological environment at the tissue level. Thus, a detailed characterization of the IVD's cellular composition informs and optimizes NPPC‐based regenerative strategies.

### 1.5 Role of Omics and Functional Integration in IVD Regeneration Research and Clinical Translation

**FIGURE 1:**
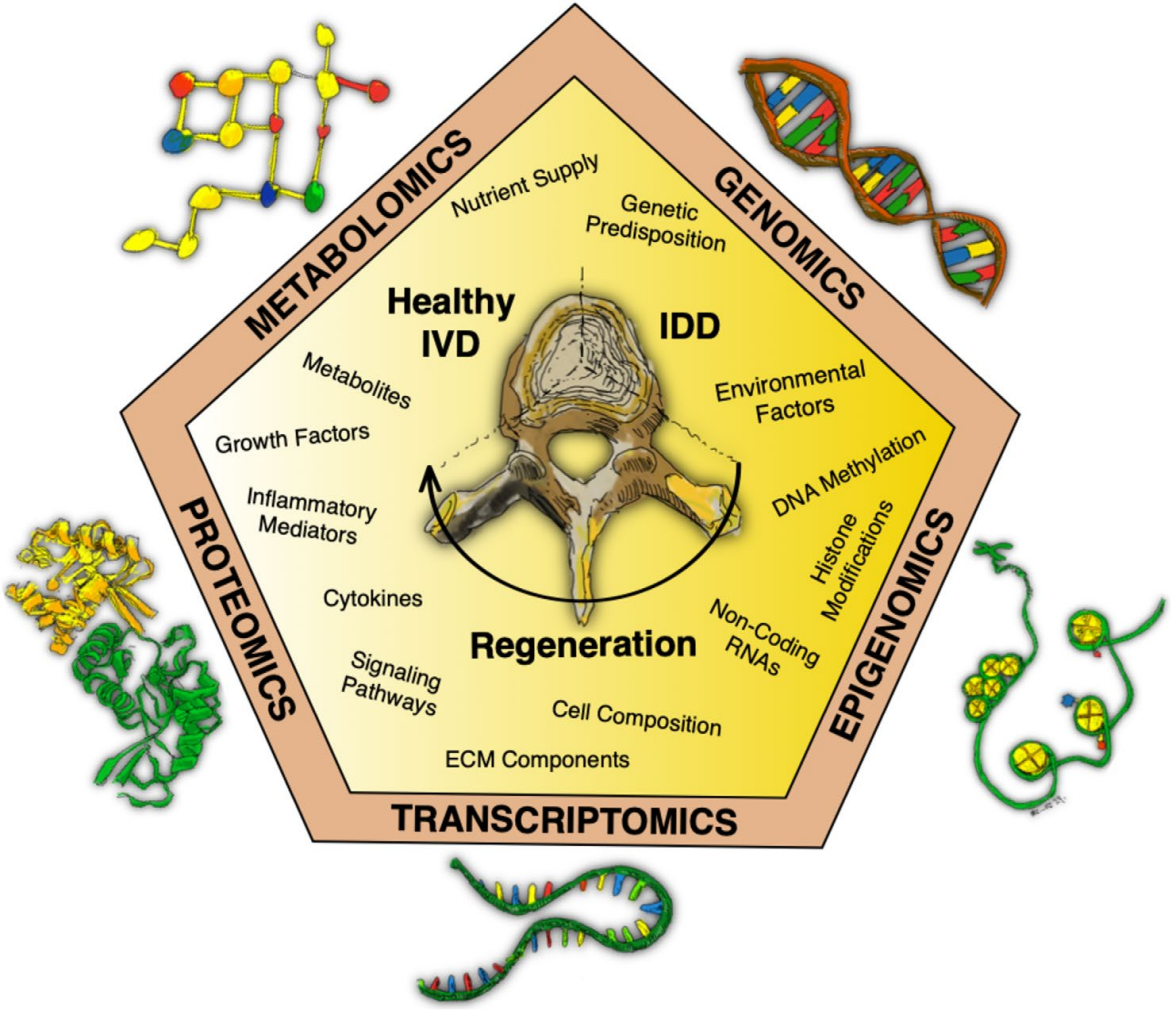
Multiple omics in intervertebral disc (IVD) regeneration research—overview of the five main omics disciplines, genomics, epigenomics, transcriptomics, proteomics, and metabolomics, and key factors within each category that influence IVD health, IVD degeneration (IDD), and potential mechanisms for regeneration.

Omics technologies allow the analysis of thousands of molecules simultaneously. Here, we focus on the five basic omics disciplines: genomics, transcriptomics, epigenomics, proteomics, and metabolomics. Genomics elucidates DNA sequence variations and genetic predispositions. Transcriptomics measures the quantity of RNA transcripts, providing a view into gene expression. Epigenomics investigates DNA and histone modifications, such as methylation, which impact gene regulation. Proteomics identifies and quantifies protein abundance. Metabolomics, quantifying metabolites, provides critical insights into cellular processes and metabolic pathways. Each discipline offers complementary insights that, taken together, illuminate the molecular networks driving IVD health and pathology (Figure 1).

Emerging omics technologies, including lipidomics, glycomics, interactomics, pharmacogenomics, metagenomics, and matrix proteomics, offer additional insight. However, these disciplines remain underrepresented or absent in IVD research, with limited available datasets, and are therefore not included in this review. Their future inclusion may further refine systems‐level understanding of disc biology and regeneration.

While omics approaches have advanced our understanding of IVD biology, key mechanisms remain only partially understood. Processes such as inflammation, ECM remodeling, and cell death involve coordinated alterations across multiple molecular layers, necessitating multiple omics integration to capture the full biological complexity. The lack of standardized strategies to integrate multiple omics and functional data in a reproducible and clinically relevant manner has limited translational progress.

To address these gaps, this review consolidates findings from transcriptomics studies with insights from other omics approaches and functional data, focusing on the intrinsic regenerative capacity of the IVD, particularly NPPCs. By synthesizing current evidence, highlighting common themes and areas where multi‐omics and functional integration are most needed, we aim to provide a roadmap for advancing IVD regeneration research and supporting the clinical translation of NPPC‐based therapies.

## 2 Deciphering IVD Biology Through Omics Approaches

The healthy IVD exhibits substantial cellular heterogeneity and relies on a balance of catabolic and anabolic processes. Cells in the IVD adapt to the hypoxic environment through anaerobic metabolism, leading to high lactate levels and low extracellular pH, regulating ECM turnover, and thereby preserving structural integrity and functionality [40, 41, 42, 43].

This balance is disrupted in IDD; ECM degradation and inflammation are increased, pH is lowered further, and ECM composition is altered. Specifically, elevated levels of collagen types I and II, alongside decreased aggrecan synthesis, contribute to these changes [44, 45, 46, 47, 48], accompanied by the upregulation of matrix metalloproteinases (MMPs) and a disintegrin and metalloproteinase with thrombospondin motifs (ADAMTs) that further disrupt ECM homeostasis [44, 46]. These processes result in reduced disc height, NP dehydration, and tissue fibrosis [45, 46, 47].

IDD is also characterized by shifts in cellular composition—most notably depletion of progenitor cells, emergence of senescent cells, and increased infiltration of immune cells [25, 49, 50]. These changes disrupt tissue homeostasis, impair regenerative capacity, and contribute to a pro‐inflammatory environment.

Key signaling pathways involved in IVD homeostasis, including Wnt signaling, TGF‐*β* signaling, and ECM‐related pathways, become dysregulated during degeneration, leading to ECM degradation and inflammation, accelerating the degenerative process. Mechanical stress and genetic predispositions amplify IDD through enhanced inflammatory pathways and increased expression of matrix‐degrading enzymes like MMPs, ADAMTs, and cathepsins [51, 52, 53]. Reduced expression of inhibitors such as tissue inhibitor of metalloproteinases (TIMPs) accelerates degeneration, particularly under mechanical overload and oxidative stress [54, 55, 56, 57, 58].

Metabolic shifts, such as reduced creatine and myo‐inositol levels alongside elevated glycine and hydroxyproline levels, indicate collagen breakdown and altered metabolism in degenerating discs [59, 60]. The accumulation of lactic acid from anaerobic metabolism acidifies the disc environment, enhancing MMP activity and accelerating collagen degradation [61]. Pro‐inflammatory cytokines, including tumor necrosis factor (TNF) and interleukin 1 beta (IL1‐*β*), further drive ECM degradation, shifting the balance toward catabolism [62].

Understanding these interactions is vital for developing therapies to restore ECM balance and improve disc health. While the precise mechanisms of IDD are still being studied, omics approaches have been central to advancing our understanding of IVD biology by enabling high‐resolution profiling. While Chapter 1.5 outlined the broader value of omics, the following sections synthesize omics‐derived insights into IVD cellular composition, biomarker expression, regulatory pathways, ECM dynamics, and the role of NPPCs in regeneration, highlighting the need for integrated multi‐omics strategies to fully elucidate the mechanisms of disc degeneration and repair.

### 2.1 Cellular Composition

Understanding the cellular foundation of the IVD's intrinsic regenerative capacity requires identifying cell types and their molecular signatures. Omics technologies—particularly transcriptomics and proteomics—have characterized cellular heterogeneity and identity.

RNAseq enables comprehensive transcriptome analysis, identifying disc‐specific markers and pathways that maintain normal tissue health or contribute to pathological changes in IDD [9]. Bulk RNAseq can provide insights into global gene expression patterns, identifying differentially expressed genes (DEG) between conditions and during IVD development and degeneration. However, pooling of cells masks cellular heterogeneity. Single‐cell (sc) RNAseq addresses this limitation by resolving transcriptomic profiles at single‐cell resolution, deconvoluting the cellular diversity, and providing a deeper understanding of the functions of subpopulations and the pathophysiological processes of IDD [63]. Spatial transcriptomics complements these methods by preserving tissue architecture and mapping gene expression within the native spatial context [64, 65], particularly relevant in IVD homeostasis, involving cell‐to‐cell signaling cascades and multiple immune pathways. Platforms like Visium and Xenium by 10× Genomics offer enhanced capabilities but still have limitations in resolution and gene panel customization [66, 67].

#### 2.1.1 Cellular Heterogeneity in Health

RNAseq has revealed substantial cellular diversity within the human IVD, identifying NP cells (NPCs), AF cells (AFCs), chondrocytes, NCs, pericytes, adipocytes, fibroblasts, stromal, endothelial, epithelial, immune, muscle, blood, and progenitor cells [28, 50, 68, 69, 70]. NPCs are the primary cell type in the NP [49, 50, 69, 71, 72], and AFCs in the AF [50, 69, 72]. Furthermore, chondrocytes, maintaining the ECM and producing cartilage matrix, and thus significantly sustaining IVD integrity [73] are a key component of the IVD's cellular landscape. They each exhibit distinct subtypes summarized in Table 1. This diversity in subtypes underscores the specialized roles these cells may play in maintaining IVD function and responding to pathological conditions. Endothelial cells and pericytes are generally involved in microvascular formation and stabilization [76, 77], although their specific roles within the IVD remain poorly understood. Adipocytes and epithelial cells provide metabolic and protective functions [78], and stromal cells offer structural support [79]. This cellular complexity reflects the presence of specialized cell types and their potential implications in the health and pathology of the IVD.

**TABLE 1:** Nucleus pulposus cell (NPCs) [49, 71] annulus fibrosus cell (AFCs) [50, 69, 72] and chondrocyte subpopulations [28, 63, 68, 70, 74, 75] identified in the intervertebral disc (IVD).

Nucleus pulposus cells (NPCs)	Annulus fibrosus cells (AFCs)	Chondrocytes
Hypertrophic chondrocyte‐like NPCs	Homeostasis AFCs	SOX9+ general chondrocytes
Effector NPCs	Inner AFCs (iAF)	Fibrochondrocytes
Homeostatic NPCs	Outer AFCs (oAF)	Fibrochondrocyte progenitors
Regulatory NPCs	Hypertropic AFCs	Cartilage progenitors
Inflammatory response NPCs	Regulatory AFCs	Homeostatic chondrocytes
Adhesive NPCs	Proinflammatory AFCs	Regulatory chondrocytes
Transient NPCs	Progenitor cells	Pre‐chondrocytes
Fibrocartilaginous NPCs		Cytokine activity‐related chondrocytes
Endoplasmatic reticulum stress NPCs		ECM maintenance chondrocytes
		Cell adhesion chondrocytes

Similar to humans, large cellular heterogeneity has been shown in bovine and murine models. Gao et al. [25] identified various NPCs in murine models, including transient, regulatory, and homeostatic types as well as NPPCs. Studies in bovine discs revealed distinct cell populations and notable heterogeneity, including NPCs, AFCs, NCs, muscle, endothelial, and immune cells [18, 27, 80, 81]. This suggests that cellular dynamics are, at least in part, conserved across species.

Moreover, RNAseq has provided evidence of the existence of progenitor cells in the NP [18, 27, 33, 68, 69, 71, 74, 82, 83], the AF [24, 74, 84, 85, 86, 87], and the CEP [24, 86, 88], where they contribute to tissue development, matrix production, growth, homeostasis, and signaling regulation [29, 89, 90]. These cells, particularly NPPCs, exhibit self‐renewal capacity and multipotency, making them promising candidates for regenerative therapies in IDD [29, 91, 92]. Markers like SOX9, GLI1, and GLI3 reflect stem cell‐related transcriptional activity [93, 94], while the presence of NC markers such as keratins, CD24, and MAP1B indicates retained regenerative and protective functions in adult IVDs [95]. The enrichment of such signatures highlights the therapeutic relevance of these cell populations in disc regeneration.

#### 2.1.2 Shifts in Cell Composition With IVD Degeneration and Aging

The cellular composition dynamically shifts during disc degeneration and aging. Transcriptomics has provided critical insights into these shifts. Tu et al. [49] noted various subtypes, including hypertrophic chondrocyte‐like NPCs, effector NPCs, homeostatic NPCs, regulatory NPCs, fibroNPCs, and adhesion‐related NPCs during degeneration. Ling et al. [71] found that metabolic homeostatic NPCs were prominent in early disc degeneration, whereas inflammatory response NPCs and fibrocartilaginous NPCs dominated the later stages. Similar shifts occur among chondrocyte subtypes during degeneration [96]. Performing developmental trajectory analysis, Jiang et al. delineated seven distinct cellular states, tracing the development from neonatal to adult cells in the IVD [69], highlighting the role of NCs present in the NP and crucial during early development. These findings demonstrate the dynamic changes in cellular composition during aging and as the disc progresses from a healthy state to more advanced stages of degeneration.

In addition to NPCs, immune and blood cells—including T cells, natural killer (NK) cells, neutrophils, myeloid‐derived suppressor cells, basophils, B cells, monocytes, and macrophages—are found in the IVD, especially under degenerative conditions. This suggests they play a role in inflammation and degeneration [49, 50, 69, 70, 97]. Increased infiltration of macrophages, T cells, myeloid progenitor cells, neutrophils, resting dendritic cells, and monocytes has been reported in degenerated compared to healthy IVDs [49, 50, 72, 98]. Additionally, heightened activation of CD4+ T cells and NK cells has been observed in IDD [70]. In contrast, eosinophils decrease in the IDD group, while T‐helper type 2 (Th2) cells and tumor‐infiltrating lymphocytes are also reduced [98, 99]. The role of dendritic cells remains unclear, with studies reporting conflicting findings—one showing activation and another indicating inhibition in IDD [70, 99]. Furthermore, monocyte and macrophage subtypes change during IDD. Effector and regulatory macrophages dominate in mild IDD, whereas the proportions of oxidative stress‐related, activated tissue, and homeostatic macrophage subpopulations increase as IDD progresses [100]. Severe degeneration features decreased homeostatic NPC markers and increased expression of adhesive and fibroNPC markers [49], indicating inflammatory shifts. Macrophage polarization towards more pro‐inflammatory M1‐like phenotypes and fewer anti‐inflammatory M2‐like phenotypes contributes to chronic inflammation that worsens degeneration [71, 101]. Certain subpopulations, such as Mɸ‐SPP1 macrophages—likely involved in inflammation and ECM degradation—are found only in degenerated discs [50]. These changes indicate a shift towards a more inflammatory microenvironment within the degenerating disc, emphasizing the importance of immune cell dynamics in IDD progression.

Another consistent finding is the progressive loss of progenitor and stem cells with IDD progression [25, 50, 63, 80, 102, 103, 104], indicating a reduction in the intrinsic regenerative capabilities of the IVD in the face of degenerative changes. This decline is particularly pronounced after age 25, with markers like TIE2 showing a substantial reduction in expression [105], and corroborates with functional assays showing a reduced chondrogenic potential in NPPCs^*CD90*+^ derived severely degenerated IVDs compared to NPPCs^*CD90*+^ derived from mildly degenerated samples [49]. These findings suggest that regenerative therapies targeting the earlier stages of IDD might be more effective due to retained regenerative capacity in younger, less degenerated IVDs.

### 2.2 Biomarkers and Cell Identity

**FIGURE 2:**
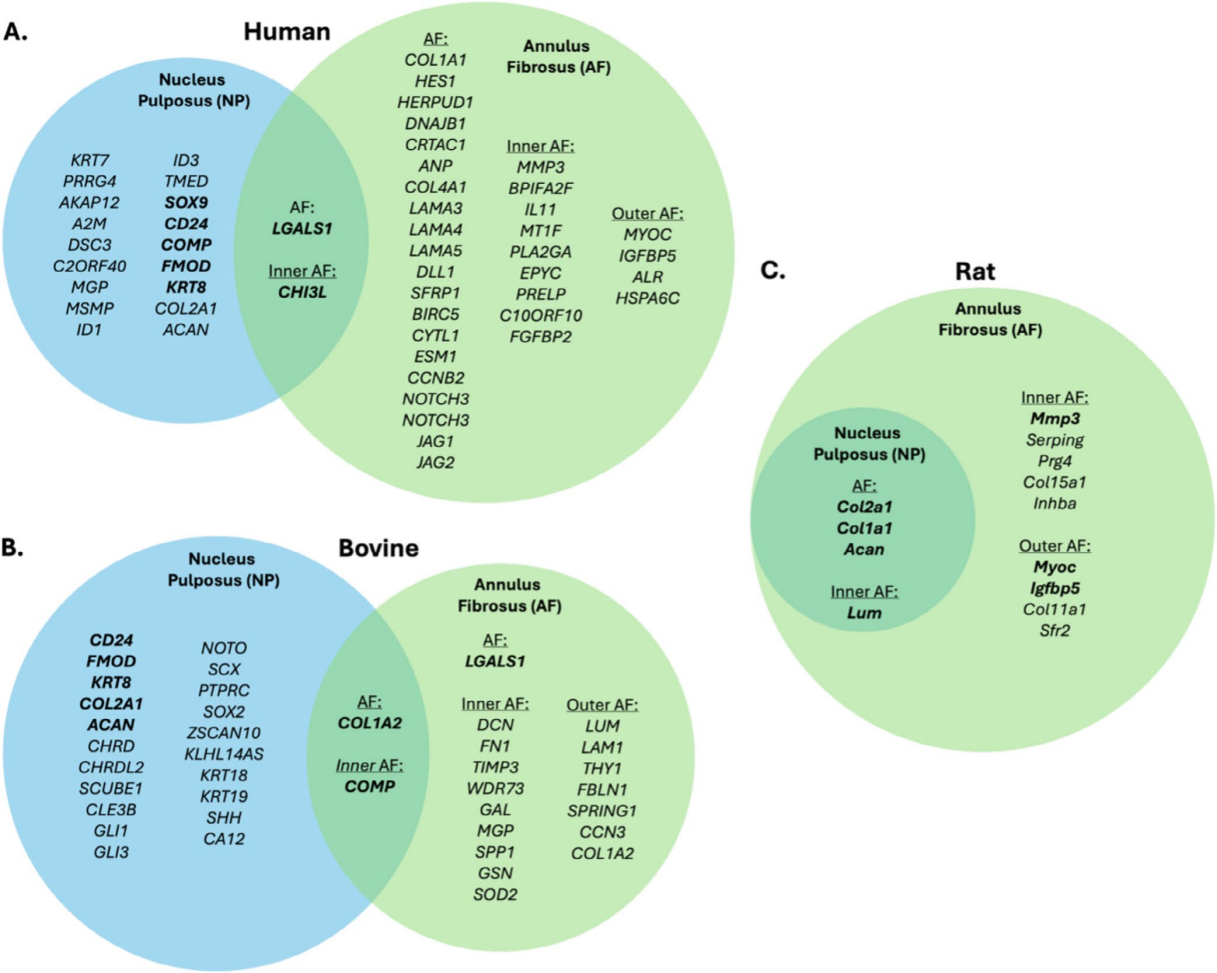
Candidate biomarkers for the nucleus pulposus (NP) and annulus fibrosus (AF) for human (A), bovine (B) and rat (C), identified through RNA sequencing [18, 27, 49, 69, 72, 80, 83, 100, 106, 107, 108, 109]. AF biomarkers are further subdivided into inner AF (iAF) and outer AF (oAF) when differentiated in the study. Biomarkers identified in more than one study are highlighted in bold. The included studies span various biological and experimental conditions, including different species, donor ages, degeneration grades, and RNA sequencing methods (bulk and single‐cell).

RNAseq has identified specific biomarkers for IVD regions, particularly the NP and AF, delineating their unique properties and roles. Figure 2 summarizes candidate biomarkers for NP and AF across human, bovine, and rat RNAseq, revealing distinctions between iAF and oAF when possible.

*ACAN* and *COL2A1* are reported in the NP of all three species [18, 49, 69, 83, 100, 107, 109, 110], indicating their fundamental roles in IVD structure and function. *COL2A1, KRT8, CD24, COMP, FMOD*, and *ACAN* were identified as NP biomarkers in both humans and bovines [18, 107], reinforcing their roles as NP biomarkers.

*COL1A1* was the most consistently identified marker gene for the AF across species [27, 50, 69, 81, 83]. Other candidate genes are *LUM*, noted in bovine [27] and rat [83]; *LGALS1* in human [111] and bovine [27]; and *MMP3, MYOC*, and *IGFBP5* in human [72] and rat [83], though findings varied among the studies.

Several genes, including *CHI3L, LGALS1, COL1A1, LUM, COL2A1, ACAN, COMP*, and *COL1A2*, have been classified variably as NP or AF biomarkers. Thus, they might instead be biomarkers for the IVD in general.

These variations could stem from inherent biological distinctions but also from varying methodologies and thresholds used in identifying these markers, as seen in the studies by Wang et al. [50] and Jiang et al. [69]. Variable factors include differences in donor characteristics, including age (ranging from neonatal to aged individuals), sex, and degeneration stage (from healthy to mildly and severely degenerated discs). Methodological differences include using bulk vs. single‐cell RNA sequencing, sample processing, culture conditions, and library preparation. Further, the choice of bioinformatic tools and analytical thresholds significantly influences marker identification. Identifying biomarkers for the NP and the AF is critical for accurately distinguishing between these tissues at a molecular level, which is essential for developing cell‐based therapies that can precisely target and regenerate the specific tissue affected by IDD.

In addition to AF and NP biomarkers, RNAseq has identified markers for other IVD‐resident cell populations, such as chondrocytes, erythrocytes, erythroblasts, pericytes, notochordal, stromal, endothelial, muscle, and immune cells, summarized in Table 2. Having biomarkers of cell populations within the IVD supports efforts to map IVD cellular heterogeneity.

**TABLE 2:** Candidate biomarkers for other IVD‐resident cell types and subpopulations identified through RNA sequencing.

Cell type	Biomarkers	References
Chondrocytes	*SOX9, ACAN, COL2A1, MIA*	[28, 63, 68, 112]
Regulatory chondrocytes	*CHI3L1, CXCL2, NFKB, CKS2, HMOX1*	[28, 49]
Effector chondrocytes	*MSMO1, HMGCS1, KLF2, CHI3L1*	[28, 49]
Homeostatic chondrocytes	*JUN, BRD2, RGS2, CCNL1, RPS29, RPS21, WSB1*	[28, 49, 109]
Notochordal cells	*KRT8, CD24, MAP1B, KRT18, KRT19*	[27, 68, 69, 81, 112]
Stromal cells	*COL1A1, COL3A1, GJA1, HES4, MMP13*	[28]
Erythrocytes/erythroblasts	*HBA1, HBB*	[50, 69, 100]
Endothelial cells	*PECAM1, CDH5, CD34*	[27, 28, 31, 50, 63, 112, 113]
Pericytes	*ACTA2, TAFLN, MCAM, MYH11*	[68, 112]
Muscle cells	*CALD1, TMP1, RBPMS, ACAT2, DES, MYH11, ACTA2, MYL9, MYLK, TAGLN, RGS5, MCAM, MYOD1, MYF5, PAX2*	[27, 28, 112]
Immune cells	*CD74, SRGN*	[27, 50, 112]

Advances in RNAseq have further led to identifying various NPPC clusters, characterized by multiple markers across different species. Identified markers include clusters of differentiation (CDs), *TEK, PDGFRA, LEPR, UTS2R, TAGLN*, *ANGP1*, and pluripotency markers. Gan et al. [68] categorized NPPCs based on their functions: NPPCs^*PAX1*+^ (ECM organization and calcium signaling), NPPCs^*ANGP1*+^ (Mitogen‐Activated Protein Kinase [MAPK] signaling and cell survival), NPPCs^*PDGFRA1*+,*PROCR*+^ (mesenchymal stem cell‐like), and NPPCs^*SOX9*+^ (proliferation and neurogenic locus notch homolog [NOTCH] signaling). Potential progenitors were also identified in bovine discs based on their high transcriptional entropy [27]. The recurrence of specific biomarkers in multiple studies and across species emphasizes their importance and relevance in NPPC biology.

Table 3 compiles NPPC markers identified from RNAseq, FACS, and related studies to provide a more comprehensive and conclusive picture of the NPPC phenotype and role.

**TABLE 3:** Recent literature on markers for nucleus pulposus (NP) progenitor cells (NPPCs) identified in the intervertebral discs (IVDs) of human and different animal models, incorporating findings from RNAseq, FACS, and other methodologies, as well as literature on the functions these markers are associated with.

Marker	Species identified	Function associated with the protein	Studies highlighting marker expression in NPPCs
*CD5, 44, 55, 70, 80*, *ITGB1* (CD24) *NT5E* (CD73), *THY1* (CD90), *ENG* (CD105)	Human, rat, mice, bovine	Cluster of differentiation markers, highlight NPPCs involvement in immune regulation, cell adhesion, and differentiation within the IVD. CD5 and CD44 are linked to notochordal cell lineages [18], CD29 and CD44 are involved in cell adhesion and signaling [114, 115], while several CD markers indicate roles in immune regulation and protection within the IVD. CD5 suggests immunomodulatory functions due to its involvement in T‐cell activation [116]. CD24 (*ITGB1*) is linked to immune regulation, protein synthesis, and extracellular matrix metabolism [50, 117]. CD55 may protect NPPCs from immune responses by preventing complement‐mediated cell lysis [118]. CD70 and CD73 (*NT5E*) contribute to immune modulation and signaling [119, 120], while CD80's role remains unclear but likely involves immune interactions in the IVD [121]. Further, CD90 (*Thy1*) is associated with stem cell properties and multipotency [49], while CD105 (*ENG*) is part of the TGF‐*β* receptor complex and plays a role in angiogenesis and cardiovascular development [122].	[18, 25, 33, 49, 71, 81, 123, 124, 125, 126]
*TEK* (TIE2)	Human, rat, mice, bovine	TIE2 (*TEK*), a cellular membrane receptor tyrosine kinase belonging to the Tie family, is primarily expressed in endothelial cells during angiogenesis [127, 128]. It mediates interactions with both the extracellular matrix and the surrounding mesenchymal stromal cells, suggesting a potential paracrine regulation [129]. TIE2, has been demonstrated to possess a critical function in maintaining NPPCs and protecting NP cells from apoptosis [29], however, the precise contribution of TIE2 to IVD homeostasis remains poorly understood.	[18, 25, 33, 105, 125, 126]
*PDGFRA*	Human, rat	PDGFRA plays a role in cell growth, differentiation, and development. PDGFRA's presence in different species underlines its importance in NPPC proliferation and differentiation.	[28, 68, 126]
*LEPR*	Human, rat	LEPR is involved in energy homeostasis and metabolism, indicating a role in the metabolic regulation of NPPCs [130].	[25, 126]
*UTS2R*	Human, mice	UTS2R is a G‐protein‐coupled receptor involved in various physiological processes, including vascular regulation and cell signaling. UTS2R is implicated in cellular responses to inflammation in the IVD [131].	[25, 126]
*SOX4*	Human, mice	SOX4 is a transcription factor, which is crucial during embryonic development and expressed in stem‐ and progenitor populations [132].	[69, 126]
*OCT4, SOX2*, and *NANOG*	Human, mice	OCT4, SOX2, and NANOG are key pluripotency markers that are typically associated with maintaining the undifferentiated state of embryonic stem cells, playing an essential role in self‐renewal and multipotency of stem cells [133].	[123]
*KRT15* (K1C15)	Bovine, rat	K1C15 is an intermediate filament protein associated with stem cells and progenitor cells; role in maintaining stemness and differentiation potential of NPPCs [134]. *KRT15* has further been associated with clusters close to notochordal cell lineages [18].	[18, 33, 126]
*TAGLN*	Mice	TAGLN is an Actin‐binding protein regulated by TGF‐*β* signaling. *TAGLN+* NP cells, capable of differentiating and populating the entire NP during development and growth, were identified in the NP but decline with aging or injury‐induced IVD degeneration, suggesting a critical role in NP formation and function [135].	[135]

### 2.3 Key Pathways in the IVD in Health and Degeneration

RNAseq not only revealed the cellular landscape and biomarkers for their identification but also identified key pathways in IVD biology. ECM‐related functions, cell adhesion, structural integrity, cellular communication, growth regulation, and angiogenesis are enriched in the IVD. Key genes such as *ACAN* and collagens underscore the role of ECM in disc structure and biomechanics [49, 69, 100, 107, 109]. Keratins and laminin subunits contribute to cell structure, stability, and ECM interactions [136], while *COMP* indicates active signaling and growth regulation [137]. These processes collectively support ECM maintenance, tissue repair, and functional resilience of the IVD.

Proteomic studies support these findings. Yang et al. [138] reported the involvement of A1AT (*SERPINA1*), A1AG2 (*ORM2*), FIBG (*FGG*), and COL1A1 in IDD regulation, and Rajasekaran et al. [139] associated degeneration with a downregulation of COL2A1 and an upregulation of COL10A1. Altered levels of collagen types I, II, and IV, fibronectin, prolargin, cartilage oligomeric matrix protein, cartilage intermediate layer protein, CILP1, HTRA1, and fibromodulin were consistently found in degenerated discs [140, 141, 142]. Similar changes were observed in chondrodystrophic canines [143], supporting translational relevance.

In addition to structural changes, numerous signaling pathways regulate IVD homeostasis and contribute to degeneration when dysregulated (see Table 4). These include Wnt, bone morphogenetic protein (BMP), NOTCH, TGF‐*β*, vascular endothelial growth factor (VEGF), hedgehog, hippo, NF‐κB, phosphatidylinositol 3‐kinase‐Akt (PI3K‐Akt), MAPK, and cytokine‐mediated pathways (IL1‐*β*, interleukin 2 [IL2], interleukin 17 [IL17], tumor necrosis factor [TNF]), as well as cell death regulators such as p53 and ferroptosis‐related signaling [18, 38, 49, 68, 71, 81, 96, 99, 107, 108, 179, 180, 181]. These cascades coordinate cell fate decisions, matrix remodeling, inflammation, and vascularization, and are central to both physiological maintenance and degenerative progression of the IVD.

**TABLE 4:** Key signaling pathways in intervertebral disc (IVD) health and degeneration as highlighted by omics disciplines. Abbreviations: NP = nucleus pulposus; ECM = extracellular matrix; IDD = intervertebral disc degeneration.

Pathway	Omics discipline	Function	IDD‐associated changes	Regenerative implication	References
Wnt	Transcriptomics, proteomics, epitranscriptomics, metabolomics	Cell fate, ECM degradation, inflammation	Dysregulated in IDD; promotes catabolism	Inhibition may aid regeneration	[39, 107, 140, 144]
TGF‐*β*	Transcriptomics, proteomics	ECM synthesis, chondrocyte proliferation and differentiation	Overactivation under mechanical stress can promote degeneration	Fine‐tuned activation supports IVD health	[112, 140, 145, 146, 147, 148, 149, 150, 151, 152, 153, 154]
BMP	Transcriptomics	Chondrogenesis, IVD development	Dysregulated expression in IDD	Enhances NP differentiation and matrix synthesis	[155]
NOTCH	Transcriptomics	Differentiation, progenitor maintenance	Altered signaling disrupts cell balance	Supports progenitor maintenance and thus a regenerative potential	[156]
Hedgehog	Transcriptomics	Cell fate, development	Impaired activity affects disc cell differentiation	May restore NP identity in degenerative context	[81]
VEGF	Transcriptomics	Neovascularization and macrophage recruitment	Promotes vascularization and inflammation in IDD	Inhibition may limit neurovascular ingrowth	[157]
PI3K/Akt	Transcriptomics, proteomics	Cell proliferation, cellular aging, oxidative stress, apoptosis, senescence	Activated under stress and lactic acid accumulation	Antioxidative and survival‐enhancing interventions	[140, 142, 158, 159]
MAPK	Transcriptomics	ECM degradation, cellular aging, inflammatory response	Mediates matrix degradation and inflammation	Targeting MAPK may reduce catabolic effects	[113, 160, 161, 162, 163]
TNF, IL1‐*β*, IL2, IL17	Transcriptomics, proteomics	Inflammatory response	Upregulated in degenerated discs	Suppression of inflammatory pathways may present a potential therapeutic strategy for IDD	[49, 140]
Hippo	Transcriptomics	Cell death, tissue regeneration, and mechanical stress responses	Dysregulated in degeneration	May regulate progenitor dynamics and repair	[164, 165]
Ferroptosis, apoptosis, necroptosis, pyroptosis, etc.	Transcriptomics	Cell death	Increased NP cell loss in IDD	Inhibition may preserve NP viability	[34, 109, 166, 167, 168, 169, 170, 171, 172, 173, 174, 175, 176, 177]
p53	Proteomics	Cell cycle arrest, apoptosis	Activated in degenerated discs	Targeting may restore NP proliferation	[142]
Advanced glycation end products	Proteomics	ECM stiffening, oxidative stress	Increased accumulation in IDD	Advanced glycation end product inhibition may preserve ECM properties	[142]
Actin cytoskeleton regulation	Proteomics	Cell adhesion, mechanotransduction	Upregulated in degeneration	Modulation may normalize mechanosensing	[140]
Viral pathway enrichment	Proteomics	Link to infection‐associated signaling (e.g., RAC1, CYCS, PSMD14)	Suggests immune‐metabolic overlap in IDD	Highlights non‐canonical pathway involvement	[178]

This complex interplay of signaling pathways governs cell fate, ECM production, inflammation, and vascularization, collectively ensuring IVD function and structural integrity. Dysregulation can lead to imbalances in cell proliferation, differentiation, and ECM integrity, driving disc degeneration. These insights emphasize the multifaceted nature of IDD and the need for integrated approaches to understand its progression and identify therapeutic targets.

### 2.4 Regulatory Mechanisms

Next to signaling pathways, epigenetic modifications, particularly DNA methylation, histone modifications, and non‐coding RNAs (ncRNA), regulate gene expression without altering the DNA sequence and are implicated in IDD [182]. DNA methylation changes have been involved in dysregulating key pathways, such as NF‐*κ*B and MAPK, in IDD [183, 184, 185], with hypomethylation of NF*κ*B pathway genes promoting inflammation and apoptosis, and hypermethylation of MAPK‐related genes reducing the expression of cell survival and repair proteins. Using transcriptomics and epigenomics, Liu et al. [186] identified ribosome activity, oxidative phosphorylation, and ECM response as major contributors in lumbar disc herniation, highlighting hub genes and associated DNA methylation sites involved in these processes.

Histone modifications, such as acetylation and methylation, further modulate chromatin structure and gene accessibility. Acetylation typically enhances transcription by loosening chromatin, whereas deacetylation and methylation condense chromatin to repress gene expression. Histone‐modifying enzymes such as SIRT6 and HDAC4 (deacetylases), and EZH2 (methyltransferase) regulate inflammatory and degenerative gene expression in IDD [37, 187, 188, 189, 190, 191]. Targeting these enzymes may offer novel therapeutic strategies for IDD.

ncRNAs, including miRNAs, lncRNAs, and circular RNAs (circRNAs), also modulate gene expression and cellular pathways both epigenomically by influencing chromatin remodeling and DNA methylation, and post‐transcriptionally by regulating mRNA stability, translation, and degradation [137, 192, 193, 194, 195, 196, 197, 198]. For instance, miRNAs and lncRNAs regulate critical cellular activities such as cell proliferation, apoptosis, ECM degradation, and cytokine release, which are pivotal in IDD progression [26, 56, 110, 199, 200, 201, 202, 203, 204, 205, 206, 207, 208, 209]. Mechanosensitive miRNAs respond to changes in matrix stiffness and tension, influencing Wnt/*β*‐catenin signaling and contributing to disc degeneration [210, 211, 212]. Similarly, lncRNAs regulate apoptosis, senescence, and ECM synthesis via Wnt/*β*‐catenin signaling, which is essential for maintaining disc integrity [80, 202, 205, 206, 208]. circRNAs can act as competing endogenous RNAs (ceRNAs), either promoting NP cell proliferation and suppressing apoptosis or facilitating IDD progression [213, 214]. Differentially expressed lncRNAs are also implicated in pathways related to osteogenic differentiation and bone remodeling [179].

Epitranscriptomic and metabolomic data further expand the regulatory landscape. Wang et al. [144] demonstrated that demethylation of LOC102555094 and upregulation of FTO and ZFP217 activated the Wnt pathway, reprogrammed glucose metabolism, and triggered PKM2 activation, linking gene regulation to metabolic and signaling changes. Xie et al. [180] identified differentially methylated mRNAs in the later stages of IDD compared to early IDD, suggesting that RNA modification contributes to disease severity by affecting RNA stability, translation, and splicing, leading to altered protein production and cellular dysfunction in the IVD. These observations underscore the relevance of epigenetic and epitranscriptomic regulation in IVD degeneration and their potential as therapeutic targets.

### 2.5 Biomarkers of IVD Degeneration

In age‐related IDD, the accumulation of senescent cells exhibiting a pro‐inflammatory phenotype perpetuates chronic inflammation, disrupts the IVD's delicate homeostasis, and accelerates degenerative changes [10]. Notably, in older individuals, there is no significant difference in the symptoms of early and late‐stage degeneration [215].

Bulk, single‐cell, and spatial transcriptomics have identified potential diagnostic biomarkers for IDD. These genes span functional categories, such as ECM regulation, cell cycle regulation, metabolic stress response, cytoskeletal integrity, and inflammation. Table 5 summarizes the identified biomarkers and their changes with IDD.

**TABLE 5:** Potential biomarkers for intervertebral disc (IVD) degeneration (IDD) progression, identified across various species using RNA sequencing (RNAseq), with biomarker changes of degenerated tissue compared to healthy IVDs (up‐ or downregulated).

Species	Tissue type	Biomarkers identified	Change in IDD	References
Human	IVD	*SPP1*	Upregulated	[112]
Human	IVD	*CYP1A1, MMP1, CCND1, NQO1*	Upregulated	[100]
Human	IVD	*FOS, JUN, JUNB, JUND, COMP, CILP, COL9A3, COL2A1, HSPA5, XBP1, HERPUD1, DDIT3*	Upregulated	[74]
Human	IVD	*CHI3L1, KRT19, COL6A, DPT, TNFAIP6, COL11A2*	Upregulated	[216]
Human	IVD	*MT1G, SPP1, HMGA1, SOD2, MT2A, UPP1, S100A2, FN1, PRG4*	Upregulated	[106]
Human	NP	*SYF2*	Upregulated	[106]
Human	AF	*MGST1, PLA2G2A, MT1F, EPYC, CHI3L1*	Upregulated	[106]
Human	CEP	*BRD4, RAF1, ANGPT1, CHD7, NOP56*	Upregulated	[70]
Bovine	NP	*ACTB, ACTG, PFN1, MYL12B*	Upregulated	[217]*
Bovine	AF	*FGF1, SPP1*	Upregulated	[217]*
Mouse	IVD	*SPP1*	Upregulated	[112]
Human	IVD	*SERPINA1*	Downregulated	[138]
Human	IVD	*MT‐CYB, MT‐ND2, SPARC, VIM, CTGF, SPTSSB, S100A1, MGP, DCN*	Downregulated	[106]
Human	NP	*C2orf40, SLPI, COL11A1*	Downregulated	[106]
Human	AF	*TAF1D*	Downregulated	[106]

*Note:* The study marked with an asterisk (*) was performed on a post‐traumatic early degeneration organ culture model that underwent one strike loading.

*SPP1* emerged as an upregulated biomarker in degenerated discs across multiple studies and species, including human, bovine, and murine models [106, 112, 217], and is implicated in cell survival, migration, and stress responses [218]. *CHI3L1* is similarly upregulated in both the NP and AF regions in humans [106, 216] and is associated with tissue remodeling, inflammation, and fibrosis [219]. In contrast, downregulation of mitochondrial and structural genes, including *MT‐CYB, MT‐ND2, SPARC*, and *DCN*, in degenerative discs [106], reflects metabolism and ECM integrity disruptions, which are IDD hallmarks.

Tissue‐specific markers reflect the spatial heterogeneity of biomarker expression in the disc. Upregulation of collagens [74, 216] may indicate a compensatory response to maintain structural integrity or a change in phenotype. Changes in the expression of cell cycle regulators, such as *RAF1*, impact cell cycle regulation and cell proliferation [220]. Metabolic stress response genes, including *SERPINA1*, highlight the metabolic challenges and stress mechanisms during degeneration [221], while inflammatory markers like *MMP1* are linked to oxidative stress and inflammation, highlighting their role in IDD [222]. Additionally, upregulation of the *AP1* transcription factor family, involved in endoplasmic reticulum stress response, illustrates the cellular stress in IDD [74]. Specifically, in the CEP, genes involved in ECM synthesis, stabilization, metabolism, and cell cycle regulation are downregulated in degenerated cells, while ECM‐degrading enzymes are upregulated [223]. These biomarkers collectively provide insights into the molecular pathways involved in IDD and may serve as targets for diagnostic and therapeutic strategies to restore disc structure and function.

Proteomic findings support transcriptomic trends. Proteomic profiling revealed differences in protein composition between young healthy discs, normal aging, and degeneration. Young discs express chondrocyte biomarkers, ECM organization, normal metabolic and hedgehog signaling proteins, such as K2C8, CD109, CHRD, and CRDL2, which are lost in aged discs [224]. Instead, aged discs express proteins associated with fibrosis and aging, including CHI3L2, A2M, and C1INH [224], alongside reduced expression of Wnt and BMP/TGF‐*β* inhibitors, suggesting a shift towards pathways that promote tissue degradation and fibrosis [224, 225]. Reanalysis of the data from Cladeira et al. [226] by Molinos et al. [227] showed an upregulation of proteins related to glycosylation and disulfide bonds with aging. Fetal IVDs express collagens such as COL15A1, linked to development and regeneration, which are absent in adult IVDs [139], alongside upregulation of proteins involved in collagen synthesis and tissue development, inhibiting cell death [228]. While aging discs exhibit increased basal expression of immune response proteins, complement inhibitors, and senescence markers, degenerated discs show higher levels of proteins associated with oxidative stress, apoptosis, and inflammation, as evidenced by upregulation of the immune response, complement activation, bacterial‐specific and host defense proteins in degenerated discs [229, 230]. The data indicate a progressive molecular shift from a regenerative to a fibrotic and inflammatory phenotype, illustrating the value of proteomics in distinguishing the stages of IVD aging and degeneration and identifying potential therapeutic targets.

Xu et al. [216] identified six key genes (*CHI3L1, KRT19, COL6A2, DPT, TNFAIP6*, and *COL11A2*) with consistent changes at protein and mRNA levels in IDD, constructing a protein‐RNA interacting network. Similarly, Yang et al. [138] found downregulation of SERPINA1 at both protein and RNA levels in IDD, identifying it as a possible biomarker and treatment target. These multi‐omics validations highlight the translational potential of integrative biomarker discovery for clinical application in IDD diagnosis and therapy.

### 2.6 Extracellular Matrix and Metabolic Shifts

ECM remodeling and metabolic dysfunction are key to elucidating IDD, as both processes are tightly linked to structural deterioration. Proteomic analyses of the IVD revealed enrichment of proteins involved in ECM‐related functions, cell junctions, and oxidative stress response. Caldeira et al. [226] observed enrichment in proteins and functions related to ECM, involved in glycosaminoglycans and carbohydrate binding, collagen organization, blood vessel and cartilage development, and glycolysis. In the NP, enrichment of proteins involved in cell junctions critical for cell–cell communication and osmoregulation, and in the AF, an enrichment of proteins for the oxidative stress response was reported [231]. This proteomic diversity underlines the functional specialization of IVD regions.

Since ECM turnover is metabolically demanding, alterations in metabolic activity are closely linked to disc degeneration. Liu et al. [186] emphasized the role of ECM remodeling in degeneration by identifying ribosome activity, oxidative phosphorylation, and ECM response as key mechanisms in lumbar disc herniation. Using an integrative transcriptomic and epigenomic approach, they highlighted hub genes and methylation sites associated with ECM function, reinforcing the importance of multi‐layered regulation in ECM homeostasis and degeneration. Complementary metabolomic studies demonstrated that IDD is associated with alterations in glycine‐serine–threonine, galactose, TCA cycle, and amino sugar metabolism, indicating global metabolic dysfunction [232].

Further studies have identified metabolic regulators relevant to disc health. Reduced phosphatidylcholine levels were determined in IDD, with a positive correlation with LPCAT1. LPCAT1 suppression reduced phosphatidylcholine production, increased NPC senescence, and induced organelle damage, suggesting its potential as a therapeutic target [233]. Similarly, Chen et al. [234] showed that overexpression of SLC43A3 could reduce lipid droplet accumulation and cell aging in IDD by inhibiting palmitic acid uptake, further emphasizing the role of metabolic regulation as a therapeutic avenue.

In terms of therapeutic modulation, Wang et al. [235] demonstrated that Sanbi Decoction, a traditional Chinese herbal formula, significantly improved serum metabolites and influenced lipid and amino acid metabolism pathways. This reduced inflammation, regulated ECM metabolic balance, and restored IVD function. These findings support the role of metabolomics in elucidating IDD mechanisms and the development of metabolism‐based regenerative therapies. In addition, Wang et al. combined RNA‐seq and metabolomics to study the effects of a fibrin hydrogel complex on tissue repair and regeneration, revealing its influence on gene expression and metabolic pathways [236]. This shows the interconnectedness of metabolic dysfunction, gene regulation, and cellular aging in disc degeneration and supports the development of metabolism‐based regenerative interventions.

### 2.7 Genetic Risk Factors

Beyond environmental and metabolic contributors, genetic predisposition also influences susceptibility to IDD. Genomics has contributed to IVD regeneration research by discovering the role of genetic variation in the susceptibility to IDD. Twin studies have established genetic variations as a significant risk for IDD, confirming the heritable nature of the condition [237]. Genome‐wide association studies (GWAS) have linked variants in collagen‐encoding genes (e.g., *COL1A1, COL9A1, COL9A2, COL9A3, COL11A1*, and *COL11A2*) and genes encoding cytokines (e.g., IL1‐*β*, IL6, and MMPs) to increased IDD risk [237]. Genetic variations have been extensively reviewed in IDD [237, 238, 239, 240, 241], highlighting their involvement in the disease's development and relevance as predictive biomarkers, determinants of individual susceptibility, and therapeutic targets. Although the precise mechanisms remain unclear, it is hypothesized that alterations in structural genes such as collagens may compromise IVD integrity, making them more prone to mechanical stress and leading to degeneration [242]. These complementary insights across molecular layers underscore the complexity of IVD biology and the necessity for integrative approaches combining multi‐omics and functional data.

## 3 Multi‐Omics Integration to Advance Regenerative Strategies

### 3.1 Importance of Multi‐Omics Data Integration

**FIGURE 3:**
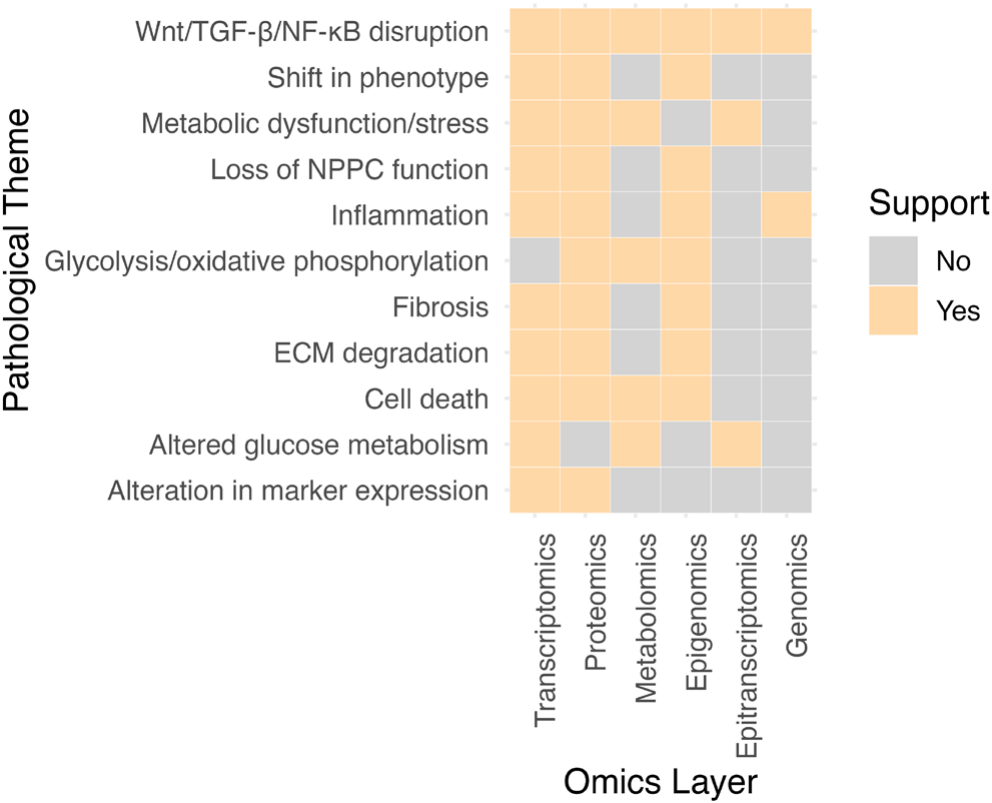
Common pathological themes emerging across omics layers. Omics layers supporting the pathological theme in orange, omics layers where the consolidated studies did not directly support the pathological theme in gray.

The number of available multi‐omics studies in the IVD field is limited. Thus, the previous chapter focused on describing findings from single‐omics studies. While these have significantly advanced our understanding of IVD biology, each omics layer captures only a part of the molecular landscape. To address this, Table 6 consolidates key findings from individual omics layers, and Figure 3 highlights recurring pathological themes across omics layers. These include ECM degradation, loss of tissue homeostasis, chronic inflammation, metabolic dysfunction, and depletion of key regenerative cell populations—particularly NPPCs—with IDD. This cross‐omics synthesis can aid in biomarker discovery, unraveling complex networks, discovering therapeutic targets, and cross‐validation of findings.

**TABLE 6:** Key insights into intervertebral disc health and degeneration derived from omics studies.

Omics layer	Healthy intervertebral disc	Degenerated intervertebral disc
Transcriptomics	Extracellular matrix maintenance and homeostasis Nucleus pulposus progenitor cells Cellular diversity Anti‐inflammatory and homeostatic gene expression	↓ Nucleus pulposus progenitor cell markers ↑ Inflammatory, fibrocartilaginous phenotype Immune cell infiltration (e.g., macrophages, T cells) ↑ Adhesive and fibrocartilaginous nucleus pulposus cells Pathway dysregulation (TGF‐*β*, NF‐κB)
Proteomics	Proteins associated with chondrogenesis and regeneration (e.g., K2C8, CD109) Normal signaling proteins Low fibrosis markers	↑ Fibrosis markers (e.g., CHI3L2, A2M) ↓ ECM proteins (COL2A1, ACAN) ↑ Proteins associated with aging, immune response, oxidative stress ↑ Glycosylation of disulfide bond modification
Metabolomics	Balance between catabolism and anabolism	↓ Creatine, glycine, TCA cycle intermediates ↑ Lactic acid ↓ Phosphatidylcholine ↑ Lipid droplet accumulation ↑ Palmitic acid uptake
Epigenomics/Epitranscriptomics	Regulated extracellular matrix synthesis, cell death and progenitor viability Balanced methylation	NF‐κB hypomethylation MAPK hypermethylation ↑ miRNAs/lncRNAs targeting Wnt/*β*‐catenin Reprogrammed glucose metabolism Histone modifications (SIRT6, HDAC4, EZH2)
Genomics	—	Variants in collagen‐encoding genes (e.g., COL9A3) Variants in cytokine‐encoding genes (e.g., IL1‐*β*, IL6, MMPs)

#### 3.1.1 Unraveling Complex Networks

Multi‐omics integration elucidates how molecular alterations cascade across regulatory layers. For example, Xu et al. [216] identified six key markers (*CHI3L1, KRT19, COL6A2, DPT, TNFAIP6*, and *COL11A*) with consistent mRNA and protein changes, constructing a protein‐RNA interaction network. Wang et al. [144] linked demethylation and metabolic shifts to Wnt activation. Liu et al. [186] identified epigenetically regulated pathways involved in ECM and oxidative phosphorylation. These findings show how cross‐layer insights uncover the multifactorial nature of IDD and its potential points of therapeutic intervention.

#### 3.1.2 Advancing Therapeutic Target Discovery

Building on this systems‐level understanding, multi‐omics analyses have facilitated the discovery of novel therapeutic targets. Downregulation of phosphatidylcholine synthesis via LPCAT1 inhibition and its impact on organelle damage and senescence suggest this enzyme as a candidate target [233]. SERPINA1, downregulated on transcript and protein levels, may serve as a diagnostic marker or treatment candidate [138]. Similarly, SLC43A3 overexpression modulated lipid droplet accumulation and slowed cell aging [234], emphasizing the role of metabolic regulation as a therapeutic avenue. In addition, Wang et al. combined RNAseq and metabolomics to study the effects of a fibrin hydrogel complex on tissue repair and regeneration, revealing its influence on gene expression and metabolic pathways [235]. Together, these findings show how multi‐omics approaches can identify and refine therapeutic strategies.

#### 3.1.3 Cross‐Validation of Findings

Integration of multiple omics data and multi‐omics enables cross‐validation across molecular layers, enhancing the robustness and reliability of research conclusions. Depending on their mode of action, genetic variants can affect different molecular levels, manifesting as changes in transcription, protein function, or metabolite processing. For instance, a genetic variant may bypass RNA transcription effects but influence protein activity or metabolic outcomes. Integrating omics data enables researchers to pinpoint where these changes are most pronounced, offering a multi‐layered validation of findings. Several studies exemplify this cross‐validation:

- COL2A1 is consistently downregulated in transcriptomic [106] and proteomic [139] datasets.
- Dysregulation of Wnt and TGF‐*β* signaling appears in transcriptomic [107], proteomic [140, 142], and epigenomic [205, 212] studies.
- SERPINA1 was identified as a potential biomarker based on consistent downregulation at both RNA and protein levels [138].

These consistent patterns across omics layers underscore the value of such integration. Bridging omics layers provides a holistic understanding of disease mechanisms, aiding the identification of robust biomarkers and therapeutic targets.

### 3.2 TEK mRNA/TIE2 Protein Discrepancy as an Example

In the case of NPPCs, the discrepancy between *TEK* mRNA and TIE2 protein levels, often observed, highlights the importance of multi‐omics approaches. Studies using techniques such as fluorescence‐activated cell sorting (FACS) and quantitative polymerase chain reaction (qPCR) have revealed frequent discrepancies between the expression of the gene *TEK* and its encoded protein TIE2. While TIE2 is present in NPPCs^TIE2+^, *TEK* is sometimes not (differentially) expressed in NPPCs^TIE2+^ compared to other NPCs. Tekari et al. [33], Guerrero et al. [91], and Sun et al. [243] all found increased *TEK* expression in bovine and human cell populations positive for TIE2, employing FACS and qPCR (see table 7 for more details). Conversely, Chen et al. [244] detected neither *TEK* mRNA expression nor TIE2 protein in mouse IVDs using spatial transcriptomics, in situ sequencing, and qPCR. Also, implementing a Cre‐recombination system to tag TIE2 fluorescently failed to visualize TIE2 protein (see Table 7 for more details). Those findings contrast with Sakai et al.'s [105] work, which has found NPPCs^TIE2+^ in murine NP tissue. Zhang et al. [245] observed no differences in *TEK* mRNA but differences at the TIE2 protein level in human NPCs, while Laagland et al. [246] were not able to detect any *TEK* mRNA but found TIE2 protein in canine NPCs under hyperosmotic conditions (see Table 7 for more details). Summarizing these recent results, the knowledge is inconclusive. While more studies have been conducted, they focus on either *TEK* expression [49, 135, 249] or TIE2 [25, 29, 35, 227, 250, 251, 252, 253, 254] only, rather than investigating both in conjunction. Broader insights could be gained by expanding the analysis beyond the IVD field.

**TABLE 7:** Overview of studies analyzing *TEK* and TIE2 expression in nucleus pulposus cells (NPCs) and nucleus pulposus progenitor cells (NPPCs) across various species, and in mouse tissue.

Author	Study summary	Species	TIE2 detection method	TEK detection method	Study result
NPC populations being positive for TIE2 and showing an increased *TEK* expression					
Tekari et al. [33]	Isolation and expansion of NPPCs^TIE2+^ for 7 days under different culturing conditions, including several growth factors and varying oxygen concentrations (21% vs. 2% oxygen).	Bovine	FACS	qPCR	Increased *TEK* in freshly isolated TIE2+ compared to TIE2‐ NPCs. Increased *TEK* in TIE2+ NPCs after 7 days of culture with FGF2 and hypoxia compared to primary TIE2‐ NPCs. Increased TIE2 in TIE2+ NPCs after 7 days of culture with FGF2 and hypoxia compared to 7 days of culture under normoxic conditions.
Guerrero et al. [91]	Isolation and expansion of NPCs in 2D or 3D cell culture and subsequent 2D culture in fibronectin coated flasks.	Human	FACS	qPCR	Increased *TEK* and TIE2 in 3D expanded NPCs compared to 2D expanded NPCs. 2D culture in fibronectin‐coated flasks showed increased TIE2 and *TEK* in NPCs previously expanded in 3D compared to 2D.
Sun et al. [243]	Culture of degenerated and non‐degenerated IVD‐derived NPCs in alginate beads.	Human	IHC	qPCR	Decrease of *TEK* and TIE2 in non‐degenerated NPCs over time.
NPC populations showing neither TIE2 protein nor *TEK* expression					
Chen et al. [244]	Characterization of NPCs and NPPCs in the mouse IVD using spatial transcriptomics and attempt of visualization of TIE2 by fluorescently tagging TIE2 with tdTomato in a Cre‐recombination system.	Mouse	tdTomato fluorescence	Spatial transcriptomics, situ sequencing, qPCR	*TEK* and TIE2 not detected in the NP.
NPC populations being positive for TIE2, but *TEK* is not (differentially) expressed					
Zhang et al. [245]	Expansion of NPCs in standard cell culture surfaces, gelatin‐coated, and ultra‐low adherence‐coated surfaces for 10 days.	Human	FACS	qPCR	Increase of TIE2+ NPCs in populations cultured on ultra‐low adherence coated surfaces compared to the other groups. *TEK* was not found to be differentially expressed between the groups.
Laagland et al. [246]	Investigation of the effect of osmolality (300, 400, and 500 mOsm/L) on the in vitro phenotype of NP cells.	Canine	IHC	qPCR	IHC showed that for higher osmolality (500 mOsm/L), TIE2 was clearly present. qPCR did not find *TEK* to be expressed in any of the experimental conditions.
TIE2 protein and *TEK* mRNA discrepancies in other contexts					
Zwiers et al. [247]	Investigation of TIE2 in endothelial cells and conduction of a Cre‐mediated knockout of TIE2 in mice.	Mouse	ELISA	qPCR	*TEK* and TIE2 expression are reduced in the knockout for the lung, heart, and aorta. *TEK* expression is reduced, but TIE2 levels are not changed for the kidney in the knockout.
	Challenge of mice with lipopolysaccharide (LPS), to mimic the initial stages of sepsis.				After LPS administration, *TEK* and TIE2 expression are reduced for lung and kidney compared to the control. *TEK* expression is reduced, but TIE2 levels are not changed for heart and liver.
Kurniati et al. [248]	TNF administration.	Mouse	ELISA	qPCR	*TEK* and TIE2 are reduced for the lung compared to the control. *TEK* expression is reduced, but TIE2 levels are not changed for the kidney.
	IL‐1*β* administration.				Reduced *TEK* mRNA in the lung, and increased *TEK* mRNA in the kidney, but no changes on TIE2 protein in both organs compared to the control.

Abbreviations: 2D, two‐dimensional; 3D, three‐dimensional; ELISA, enzyme‐linked immunosorbent assay; FACS, fluorescence activated cell sorting; FGF2, fibroblast growth factor 2; IHC, immunohistochemistry; IL‐1*β*, interleukin 1 beta; IVD, intervertebral disc; LPS, lipopolysaccharide; NP, nucleus pulposus; NPCs, nucleus pulposus cells; NPPCs^TIE2+^, TIE2+ NPPCs; qPCR, quantitative polymerase chain reaction; TNF, tumor necrosis factor.

This discrepancy between *TEK* and TIE2 was also detected in other cells and tissues. Zwiers et al. [247] and Kurniati et al. [248] both found *TEK* expression levels and TIE2 protein levels to differ in some of the mice tissues they investigated but align in others (see Table 7 for more details). Although not directly relevant to IVD research, those studies highlight that mRNA and protein expression levels must not correlate in the context of *TEK* and TIE2.

The inconsistencies in findings across studies, species, and tissues remain unclear, thus raising the question: What factors contribute to these discrepancies? Possible explanations include methodological differences (e.g., variation in primers, antibodies, or FACS gating strategies), species‐specific differences, or the complex regulation of TIE2. Similar discrepancies observed outside of IVD research suggest that post‐transcriptional or post‐translational regulation likely contributes to the difference between mRNA (*TEK*) and protein (TIE2) levels, alongside methodological and interspecies differences. Notably, the human *TEK* gene undergoes post‐translational modification into at least five different protein variants [255], so‐called isoforms present in different tissues, which may contribute to variance in detection efficiency.

The review of the available literature underscores the need for future studies to measure both *TEK* and TIE2 simultaneously—where cell number and experimental design permit—before progressing towards more clinically relevant approaches. While combining FACS and qPCR would increase meaningfulness, integrating proteomics and transcriptomics could offer a more holistic view of *TEK/TIE2* regulation. Incorporating additional omics layers, such as epitranscriptomics, would further refine this understanding, highlighting the potential of multi‐omics approaches in advancing IVD regeneration research.

### 3.3 Limitations of Mult‐Omics Integration and Future Needs

In this review, we primarily consolidated findings from single‐omics studies and illustrated how even limited multi‐omics studies can help unravel molecular connections, validate findings across data layers, and support therapeutic development. While this synthesis offers a more holistic view than single‐omics alone, the integration of primary data across omics layers would be necessary to gain a more interconnected, mechanistic understanding of IDD. However, direct multi‐omics integration still faces several major obstacles.

One major obstacle is the limited availability of high‐quality healthy human IVD tissue, which limits sample size and statistical power. Moreover, inherent sample heterogeneity due to varying anatomical levels, grades of degeneration, or patient comorbidities introduces confounding variability and complicates data interpretation.

Transcriptomics technologies are susceptible to batch effects, sample preparation, and cell isolation methods, which often introduce inconsistencies, complicating data interpretation [6]. Factors such as 2D versus 3D culture, normoxic vs. hypoxic conditions, and prolonged in vitro expansion alter the transcriptome, leading to artifacts that compromise biological interpretation and reduce reproducibility across datasets.

Multi‐omics integration adds further complexity. Each omics type has distinct noise levels, detection sensitivity, and data structure, requiring advanced integration strategies [256]. Early, intermediate, and late integration strategies differ in how they combine data, with intermediate integration often preferred for its balance between information preservation and computational feasibility. Tools like MOFA, SNF, and DIABLO help identify subtypes and functional modules, while pathway‐informed models like PathME improve interpretability [257]. Tools like GLUE and MOMIC provide scalable integration and regulatory inference, whereas knowledge‐guided models enhance robustness and interpretability [258, 259, 260]. Despite recent advances in the availability of such tools, there is no standardized pipeline or consensus on best practice for IVD‐specific multi‐omics integration. Key challenges remain, including metadata harmonization, managing heterogeneous and sparse datasets, modeling non‐linear feature interactions, and developing annotation standards [257, 261, 262].

Biological, technical, and ancestral biases further limit generalizability. Approaches like PhyloFrame demonstrate how integrating population structure can enhance predictive modeling and reduce overfitting in underrepresented subgroups [263]. IVD research would benefit from similar frameworks that account for age, sex, and degeneration stage‐specific variability.

To enable clinical translation, omics data must be integrated into predictive models for patient stratification and early biomarker discovery. These models require validation across large, standardized, multicenter cohorts [264].

Future efforts should focus on protocol and metadata standardization and developing integrative computational frameworks suited for the IVD microenvironment. Incorporating spatial, biomechanical, and temporal data, reducing cell culture and batch‐induced artifacts, and applying artificial intelligence‐guided, knowledge‐informed models will be key. These approaches will help realize the promise of multi‐omics in IVD regeneration research.

### 3.4 Beyond Data: Standardizing Terminology

In addition to standardizing and improving multi‐omics data integration, achieving coherence in nomenclature is an important yet often overlooked factor for consistency, clarity, and understanding in IVD regeneration research. Standardized terminology facilitates accurate communication, especially when distinguishing between gene‐ and protein‐level findings.

This review follows the “Guidelines for Human Gene Nomenclature” by Bruford et al. [265] for gene names and symbols, and the “Protein Nomenclature Guidelines” jointly developed by the European Bioinformatics Institute (EMBL‐EBI), the National Center for Biotechnology Information (NCBI), the Protein Information Resource (PIR), and the Swiss Institute for Bioinformatics (SIB) [266] for proteins. Conventions allow the use of official gene and protein symbols without prior mention of their full names. Furthermore, the guidelines recommend using uppercase letters, with symbols italicized for genes and in regular font for proteins. For most vertebrates including bovine, the same suggestions apply, whereas for rodent proteins symbols are also all uppercase letters in regular font for proteins, whereas gene symbols are the first letter uppercase, remaining letters lowercase in italicized punctuation. This review aims to ensure precision and clarity in presenting multi‐omics data and its implications for IVD research by adhering to these established guidelines.

In the literature, inconsistencies exist in *TEK*/TIE2 nomenclature. Although “TEK” is the official name for both gene and protein [265, 266], its interchangeable use contributes to ambiguity when distinguishing between gene and protein levels. Sakai et al. [29] introduced the widely adopted term “Tie2+ NPPCs” to denote TIE2‐expressing cells. To promote clarity, we suggest keeping “TIE2” (capitalized) as the standard name for the protein in the context of IVD regeneration research, to more closely adhere to protein nomenclature guidelines [265, 266], while continuing to use “*TEK*” (respectively “*Tek*” for rodents) for the gene. This distinction would aid in clarifying discussions of gene versus protein‐level findings and enhance interpretability when integrating transcriptomic and proteomic data in multi‐omics research. While this review focuses on RNA sequencing and multi‐omics contributions to IVD regeneration, addressing this nomenclature issue is critical for ensuring consistency and accuracy in interpreting and communicating *TEK*/TIE2 data and beyond.

## 4 Translational Challenges and Clinical Considerations Towards Leveraging the Intrinsic Regenerative Capacity of the IVD Using Nucleus Pulposus Progenitor Cell‐Based Therapies

Translating NPPC‐based therapies into clinical practice involves overcoming several critical challenges. These include standardizing protocols for cell isolation and expansion, ensuring safety and efficacy, maintaining cell phenotype and niche regulation, developing effective delivery methods, functionally validating their regenerative capacity, translating findings from animal models to humans, and addressing regulatory and ethical considerations.

The consolidation of omics findings presented in the previous chapters of this review provides a foundation to support and guide these efforts. Omics‐derived markers enable NPPC identification. Insights into the molecular mechanisms of NPPC depletion, inflammation, and ECM degeneration highlight strategies to preserve NPPC phenotype and function, such as modulating inflammation or restoring ECM composition. Moreover, understanding key signaling pathways suggests targets for preconditioning and modulation. Transcriptomic and proteomic data further inform the design of delivery systems such as ECM‐mimicking hydrogels that preserve cell function and support engraftment. Metabolomic and epigenomic insights inform co‐delivery strategies using growth factors and extracellular vesicles (EVs). Addressing these challenges is crucial for the successful clinical translation of NPPC‐based therapies. A key initial step in this process involves the standardization and optimization of cell isolation.

### 4.1 Standardization of Protocols

Isolation methods for NPPCs^TIE2+^ yield populations with variable purity and expansion capacity [35]. Techniques such as enzymatic digestion, FACS, magnetic‐activated cell sorting (MACS), differential adhesion, pluriSelect, and related approaches typically yield 1%–5% NPPCs^TIE2+^ [267]. Earlier studies reported NPPCs^TIE2+^ populations ranging from approximately 8% to 13% [33, 268]. However, recent findings suggest considerable variability from these previously reported ranges, indicating a need for revised standards and rigorous validation studies [269].

While FACS provides higher sorting efficiency [35], it requires fluorescent labeling, which may alter cell biology. MACS and pluriSelect, although less invasive and cost‐intensive, still rely on antibody labeling and demonstrate lower sorting efficiencies [35], underscoring the urgent need for efficient, effective, and label‐free sorting technologies. Such advancements would significantly enhance NPPC purity, viability, and regenerative potential, facilitating clinical translation. Cryopreservation protocols have maintained NPPC viability around 90%, yet consistency across laboratories remains challenging [268].

Further, culture conditions contribute to variability, with standard 2D culture commonly used, alongside specialized conditions such as ECM–coated substrates, spheroid colony formation, or low‐density culture methods [270, 271, 272]. These approaches directly influence NPPC behavior, impacting colony formation, self‐renewal, and multipotency [273]. In vitro studies have consistently demonstrated differentiation potential towards chondrogenic, adipogenic, osteogenic, and occasionally neurogenic lineages, which correlate with regenerative performance in vivo [267]. Animal models have confirmed improvements in disc height, tissue architecture restoration, and reduced degeneration following NPPC transplantation [25, 273]. However, variability across isolation and expansion methods remains a barrier to reproducibility and reliable clinical outcomes.

Notably, discrepancies between *TEK* and TIE2 expression highlight the need for unified criteria in progenitor cell identification. Reliance on either transcript or protein alone may result in misclassification, underscoring the importance of multi‐level validation to ensure accurate and reproducible cell characterization across studies.

Standardizing NPPC isolation and expansion protocols is thus essential to ensure consistent therapeutic efficacy. Without standardized good manufacturing practice (GMP)‐compliant protocols, regulatory approval may be compromised, delaying clinical translation. Implementing unified, validated protocols will increase reproducibility, enhance safety, and accelerate NPPC therapies' translation from bench to bedside [35, 269].

### 4.2 Ex Vivo Phenotype Preservation

Maintaining NPPCs' unique phenotype during ex vivo expansion is crucial for therapeutic efficacy. Key phenotypic markers include surface proteins such as TIE2, CD24, GD2, CD105, and CD90, and functional markers like aggrecan and type II collagen. These markers consistently correlate with NPPC multipotency, colony formation capability, and regenerative performance in vivo [29, 274, 275]. Culture conditions mimicking the native NP environment, such as hypoxic conditions (2%–3.5% O_2_), supplementation with specific growth factors like FGF2 and Ang‐1, and culture on substrates resembling the ECM, are essential for preserving these phenotype‐defining features [33, 34, 276, 277, 278]. In vitro cell culture experiments have demonstrated that hypoxia, bFGF, laminin, 3D culture systems, and whole tissue culture conditions are all beneficial for maintaining the NPPC^TIE2+^ phenotype [33, 91, 245, 254, 268]. Moreover, cellular heterogeneity suggests potential synergistic interactions, indicating that co‐culture systems may enhance NPPC expansion in vitro, better mimicking in vivo conditions for effective regeneration. Standardizing phenotype‐preserving culture conditions will help retain the regenerative potential of NPPCs, directly enhancing clinical outcomes by ensuring transplanted cells closely mirror native physiology and regenerative functions.

### 4.3 Cell Survival and Phenotype Preservation in Vivo

Enhancing NPPC survival within the degenerative disc microenvironment is essential for therapeutic success. The harsh microenvironment of degenerated IVDs—characterized by low oxygen tension (hypoxia), a drop in pH, low nutrient availability, altered matrix composition, ECM degradation, and elevated proinflammatory cytokines—significantly compromises NPPC viability, proliferation, and therapeutic potential post‐transplantation [29, 279]. To date, the limiting factors for successful cell therapy include mechanical stress during delivery, ECM disruption, the harsh microenvironment of degenerated discs, and host inflammatory response. Thus, modulating inflammation and restoring niche homeostasis through immunomodulatory agents may be necessary to harness the regenerative potential of NPPC^TIE2+^ in vivo. Co‐delivery with protective biomaterials or bioactive agents may further support engraftment and integration. Additionally, pre‐conditioning strategies, such as hypoxia and pulsed electromagnetic fields (PEMFs), have shown potential in improving cell viability and ECM production.

#### 4.3.1 Delivery Methods and Niche Modulation

Developing minimally invasive, effective delivery systems is crucial for translating NPPC therapies into clinics. Injectable, functionalized hydrogels represent promising solutions, offering high cell viability (> 90%), improved cell retention, and enhanced ECM synthesis, demonstrated by upregulated NP‐specific genes such as KRT8, KRT18, and FOXF1 and elevated aggrecan and type II collagen production [29, 274, 275].

Hydrogels, including nanohybrid peptide formulations, laminin‐functionalized polyethylene glycol, and collagen–hyaluronic acid matrices, have mechanical properties close to native NP tissue stiffness. These hydrogels demonstrate promising in vivo outcomes, such as maintained disc height (87% disc height index (DHI) at 8 weeks) and restored IVD biomechanical properties [280, 281, 282]. Thus, hydrogel‐based delivery systems combining targeted cellular support and mechanical reinforcement represent key advancements for clinical NPPC delivery and functional integration [283].

Delivery in hydrogels composed of ECM‐mimicking materials such as hyaluronic acid, alginate, agarose, or decellularized ECM can support NPPC survival. These scaffolds reduce mechanical stress during injection, preserve cell–cell and cell‐ECM interactions, and provide a protective barrier [284, 285, 286, 287]. Moreover, hydrogels allow a sustained release of bioactive molecules, directed delivery, and immune shielding, while supporting mechanical properties by mimicking the ECM [287]. Incorporation of growth factors can further promote cell survival, differentiation, and tissue regeneration [284, 285].

To mimic the in vitro findings, the NPPCs could be injected alongside a hydrogel or substrate functionalized with bFGF or laminin. Using a hydrogel recreates the beneficial 3D conditions explored in vitro. Speer et al. [288] investigated the effect of a polyethylene glycol hydrogel functionalized with laminin on NPCs and demonstrated that their scaffolds could induce regenerative behavior. Hydrogels, combined with antacids, could increase the acidic pH of the degenerated disc environment, as explored by Gansau et al. [289]. Additionally, several studies have found that injecting anti‐inflammatory drugs into the disc, partially combined with hydrogels, reduced inflammation and slowed disc degeneration [290, 291]. This further modifies the IVD microenvironment towards a more favorable environment for the NPPC^TIE2+^.

Other efforts have focused on delivering bFGF to the disc. For instance, Liang et al. [292] explored bFGF‐containing polymeric microspheres for potential co‐delivery with mesenchymal stem cells (MSCs) to the IVD. However, the data collected on bFGF's effect on the disc have been partially contradictory. bFGF might play different roles depending on various stages of degeneration, and its administration could potentially induce undesirable or unexpected effects [293].

Another promising niche modulation involves the Ang‐1/TIE2 signaling pathway. Sakai et al. [29] reported that Ang‐1, a ligand of TIE2, could stimulate proliferation to NPPC^TIE2+^ in vitro. However, a study by Bischof et al. [294] could not replicate those findings. This underscores the complexity of this interaction and leaves a need for further investigation. While Ang‐1 and TIE2 interact and may be important regulators of NPPC^TIE2+^, they are also involved in angiogenesis [295]. This raises concerns about unforeseen reactions and consequences when targeting this pathway in the already degenerated and vascularized IVD [279]. Besides the Ang‐1/TIE2 pathway, the modulation of pathways involved in IDD, recapitulated in chapters 2 and 2.3, could also be interesting for restoring the NPPCs' niche.

Lastly, Otani et al. [252] demonstrated that the direct injection of NPCs in a 0.5% sodium hyaluronate solution into a rat model of IVD degeneration maintained the DHI. However, histological analyses did not show a clear improvement upon administration of the NPCs. This demonstrates that direct modulation of the niche might not necessarily be needed, but its modulation could further help improve the regenerative outcome.

Similarly, hypoxic conditions necessitate active signaling pathways such as TIE2/Ang‐1 or ECM cues (e.g., small leucine‐rich proteoglycans) for sustained cell survival [90, 296]. Additionally, ECM stiffness and composition, particularly supportive ECM proteins like tenascin‐C, significantly impact NPPC differentiation and regenerative efficacy [25, 297].

Co‐delivery of NPPCs with EVs represents another promising strategy to enhance the regenerative potential of NPPCs due to their anti‐inflammatory, neuroprotective, and regenerative properties [284, 285, 286, 287]. EVs carry bioactive cargos such as mRNAs, miRNAs, and proteins that modulate the microenvironment by suppressing inflammation, inhibiting apoptosis, and ECM degradation, promoting autophagy, and enhancing cell proliferation and signaling [287].

#### 4.3.2 Pre‐Conditioning

Next to niche modulation, pre‐conditioning cells before implantation could be another strategy to handle the harsh environment at the transplantation sites. Pre‐conditioning donor cells before transplantation can enhance their resilience to hypoxic stress, for example, by making cells more resistant to cell death stimuli. Exposure to stressors, such as heat shock, hypoxia, or nutrient deprivation, activates pro‐survival pathways and improves adaptation to the harsh microenvironment. Pharmacological agents may further enhance these effects [284].

The harsh microenvironment of degenerated IVDs significantly compromises NPPC viability, proliferation, and therapeutic potential post‐transplantation [8, 23]. Studies have demonstrated that acidic conditions (approximately pH 6.8) induce apoptosis, ferroptosis, and pyroptosis in NPPCs [223]. However, preconditioning cells (e.g., polyethylene glycol‐polyisobutylene [PEG‐PIB] treatments) has shown promise in mitigating pyroptosis and enhancing survival [223].

PEMFs have demonstrated beneficial effects on IVD cells by modulating inflammation, promoting matrix synthesis, and activating protective signaling pathways. PEMFs enhanced ECM gene expression in degenerated NPCs and activated the SIRT1‐autophagy axis [298]. In bovine NPCs exposed to an inflammatory environment, PEMFs reduced IL‐6 expression and blocked IL‐1α‐induced activation of NF‐κB and p38‐MAPK signaling [299]. Similarly, PEMFs reduced the expression of catabolic and inflammatory genes such as IL‐6, MMP2, MMP13, and NF‐κB in human NP and AF under proinflammatory stimulation [300]. Further matrix synthesis is enhanced by PEMFs via BMP pathways [301]. In vivo application of PEMF stimulation directly to a rat model reduced IDD, underscoring its regenerative potential [298].

HIF1A (hypoxia‐inducible factor 1 α) has been demonstrated to play a protective role in NPPCs exposed to mechanical loading by enhancing autophagy. HIF1A expression is diminished in degenerated human and rat IVDs, correlating with reduced NPPCs and increased apoptosis. In vivo transplantation of HIF1A‐overexpressing NPPCs into compressed rat discs enhanced cell survival, reduced apoptosis, increased ECM synthesis, and delayed disc degeneration [302]. This makes it a promising target for improving NPPC‐based regenerative therapy.

Ultimately, the regenerative potential of these strategies must be functionally validated to ensure translational relevance. Thorough preclinical studies assessing NPPC performance and survival in conditions mimicking clinical scenarios are indispensable before clinical translation, ensuring safety and therapeutic effectiveness [303].

### 4.4 Functional Validation of NPPC Regenerative Capacity

The current evidence that these NPPCs are potentially regenerative and can activate existing cells residing in the tissue comes from in vitro and in vivo studies. In organ culture experiments using MSCs and homing behavior into healthy bovine IVD explants, it was shown that TIE2+ cells were increased after MSC homing [304]. Furthermore, a recent study on the EV's potential isolated from TIE2‐positive NPCs (NPC^TIE2^ ^+^) could show a regenerative effect in a degenerative rat needle puncture‐induced coccygeal IDD model [30]. The study found that NPC^TIE2^ ^+^‐derived EVs were superior to MSC‐derived EVs regarding regenerative outcomes in the in vivo application. In vitro, the NPC^TIE2^ ^+^‐derived EVs were efficiently internalized by degenerative NPCs, enhanced cell proliferation, and reduced senescence. A single injection of NPC‐derived EVs preserved DHI, attenuated degenerative changes, and notably reduced mechanical hypersensitivity [30]. Interestingly, DHI was recovered compared to sham in both human NPC^TIE2^ ^+^‐derived and NPC^TIE2^ ^−^‐derived EVs compared to human bone‐marrow MSC‐derived EVs.

Despite promising preclinical evidence, clinical progress in disc regeneration has been slow, with many trials not reaching completion. Ambrosio et al. [305] analyzed the publication status and funding sources of clinical trials investigating cell‐based treatments for chronic LBP due to IDD. They found that less than 30% of trials reached publication, with high dropout rates, small sample sizes, and heterogeneous patient selection contributing to premature termination. While no significant impact of funding source was observed, high costs and translational challenges were substantial barriers. Their findings highlight the need for sustained financial support and strategic trial design to overcome the translational hurdle from bench to bedside of NPPC‐based therapies.

Nevertheless, progress continues, as randomized double‐blinded clinical trials are currently ongoing or have been completed. The NCT03955315 trial at Tokai University Hospital and collaborating centers investigates the intradiscal injection of “discogenic” cells combined with hyaluronic acid as a carrier [306]. In parallel, the NCT03347708 trial by DiscGenics Inc. in the United States evaluates the safety and efficacy of “discogenic progenitor cells” [307]. As of now, no results have been published.

Several patent applications by DiscGenics Inc., including WO2011122601A1 and US11168305B2, comprise the usage and culture of IVD‐derived NPPCs that can be used in the therapy of IVD injury [308, 309]. The NPPCs are isolated from the NP of a vertebrate and characterized by being positive for at least one surface marker from among TIE2 and GD2. Both recent patents on TIE2+ cell expansion using spheroids and cryo‐protection of the NPPC^TIE2^ ^+^ project have been filed and provide evidence for clinical relevance [29, 310]. These clinical studies will provide direct evidence for regenerative potential.

### 4.5 Regulatory and Ethical Considerations

Clinical translation of NPPC therapies demands compliance with stringent and diverse regulatory frameworks, varying significantly between regions. In the United States, these therapies must adhere to Food and Drug Administration (FDA) guidelines for human cells, tissues, and cellular and tissue‐based products (HCT/Ps). European regulations classify NPPC‐based treatments as advanced therapy medicinal products (ATMPs), requiring rigorous GMP compliance and clinical validation [311, 312]. Meanwhile, Japan's accelerated approval process facilitates rapid clinical application but introduces additional safety and equity concerns due to shortened evaluation periods [313]. Given the variability across regulatory environments, international harmonization of standards and approval criteria has been strongly advocated to streamline global translation and adoption of NPPC‐based therapies [312].

Ethically, robust clinical translation necessitates meticulous patient‐informed consent processes that transparently communicate potential risks and benefits. Vulnerable patient populations must be protected, data privacy ensured, and adverse events systematically documented and monitored [297, 314]. Equitable access represents another crucial ethical dimension; geographical disparities and high treatment costs may otherwise limit therapeutic accessibility [111]. Addressing these ethical concerns proactively will foster public trust, ensure compliance with ethical standards, and facilitate the broader acceptance and clinical implementation of NPPC therapies.

### 4.6 Clinical Implications

It is imperative to address these translational challenges systematically—through standardized cell isolation and expansion, adaptive strategies to improve survival in harsh disc conditions, preservation of cell phenotype, optimized hydrogel‐based delivery methods, and comprehensive regulatory and ethical compliance. Overcoming these barriers will ensure consistent, safe, effective, and broadly accessible NPPC‐based therapies, ultimately providing significant clinical benefits for patients suffering from IDD.

## 5 Conclusion

In conclusion, omics approaches have advanced our knowledge of NPPCs and IVD biology. RNAseq has uncovered substantial cellular heterogeneity, enabled biomarker discovery, and revealed dynamic shifts in cell composition, gene expression, and signaling pathways, such as Wnt and TGF‐*β*, across IVD development, aging, and degeneration. Complementary omics layers have contextualized these findings, elucidating metabolic, proteomic, and epigenetic alterations associated with IDD.

This review highlights the value of consolidating findings across omics layers, while also emphasizing the need for future studies directly integrating primary multi‐omics data. Such integration is essential to resolve discrepancies—such as those observed between *TEK* mRNA and TIE2 protein levels, to drive the discovery of novel therapeutic targets, and to pave the way for precision and personalized medicine in the treatment and prevention of IDD. Looking ahead, multi‐omics and functional integration are needed to clearly define and validate NPPC identity, map the niche and interactions, and link the different molecular layers for a comprehensive system‐level understanding.

Also, translating NPPC‐based therapies into clinical applications remains challenging, requiring standardized isolation and expansion protocols, maintenance of phenotypic stability, optimization of delivery methods, and regulatory compliance. Omics‐informed insights into the IVD microenvironment may facilitate the development of combinatorial approaches such as co‐delivery of cells with niche modulators to enhance therapeutic efficacy.

Overcoming these barriers is necessary to fully harness the NPPCs' regenerative potential and bridge the gap between multi‐omics discoveries and clinical practice. Achieving this holistic understanding of IVD biology will ultimately drive the development of effective, patient‐centered strategies to restore disc function and improve clinical outcomes.

## Author Contributions

Anja Stirnimann and Fabian Ille conceptualized and defined the outline of the review. Anja Stirnimann authored most of the review. Leon Schlagenhof contributed the section on discussing *TEK*/TIE2 divergence in NPCs at mRNA and protein levels. Benjamin Gantenbein drew and designed Figure 1 and contributed Section 4.4. Fabian Ille contributed the section on translational challenges. Section 4.3 was co‐authored by Fabian Ille, Leon Schlagenhof, and Anja Stirnimann. Fabian Ille and Benjamin Gantenbein provided funding, infrastructure, and supervision. The manuscript was thoroughly reviewed and edited by Anja Stirnimann, Fabian Ille, Benjamin Gantenbein, and Leon Schlagenhof.

## Conflicts of Interest

Benjamin Gantenbein, PhD, is a member of the editorial board of JOR Spine.
